# The Nonmydriatic Fundus Camera in Diabetic Retinopathy Screening: A Cost-Effective Study with Evaluation for Future Large-Scale Application

**DOI:** 10.1155/2016/4625096

**Published:** 2016-11-03

**Authors:** Giuseppe Scarpa, Francesca Urban, Stela Vujosevic, Michele Tessarin, Giovanni Gallo, Annalisa Visentin, Emanuela Foglia, Lucrezia Ferrario, Edoardo Midena

**Affiliations:** ^1^Department of Ophthalmology, Ca Foncello Hospital, ULSS 9 TREVISO, 31100 Treviso, Italy; ^2^Department of Ophthalmology, University of Padova, 35122 Padova, Italy; ^3^General Management, Ca Foncello Hospital, ULSS 9 TREVISO, 31100 Treviso, Italy; ^4^Primary Care Physicians Coordinating Body, Ca Foncello Hospital, ULSS 9 TREVISO, 31100 Treviso, Italy; ^5^Centre for Research on Health Economics, Social and Health Care Management (CREMS), LIUC-Università Cattaneo, 21053 Castellanza, Italy; ^6^Fondazione G. B. Bietti, IRCCS, Roma, Italy

## Abstract

*Aims*. The study aimed to present the experience of a screening programme for early detection of diabetic retinopathy (DR) using a nonmydriatic fundus camera, evaluating the feasibility in terms of validity, resources absorption, and future advantages of a potential application, in an Italian local health authority.* Methods*. Diabetic patients living in the town of Ponzano, Veneto Region (Northern Italy), were invited to be enrolled in the screening programme. The “no prevention strategy” with the inclusion of the estimation of blindness related costs was compared with screening costs in order to evaluate a future extensive and feasible implementation of the procedure, through a budget impact approach.* Results*. Out of 498 diabetic patients eligible, 80% was enrolled in the screening programme. 115 patients (34%) were referred to an ophthalmologist and 9 cases required prompt treatment for either proliferative DR or macular edema. Based on the pilot data, it emerged that an extensive use of the investigated screening programme, within the Greater Treviso area, could prevent 6 cases of blindness every year, resulting in a saving of €271,543.32 (−13.71%).* Conclusions*. Fundus images obtained with a nonmydriatic fundus camera could be considered an effective, cost-sparing, and feasible screening tool for the early detection of DR, preventing blindness as a result of diabetes.

## 1. Introduction

With the increase in life expectancy, there is, proportionately, a dramatic increase in age-related health conditions including, in particular, eye diseases. Among all the eye pathologies, diabetic retinopathy (DR) is currently one of the leading causes of blindness in the working-age population in developed countries [1–3]. Epidemiological data show that the prevalence of DR is around 30% in the diabetic population and that the annual incidence varies from 2% to 6% [4]. Every year, up to 1% of diabetic patients develop serious ocular complications leading to both poor quality of life and a socioeconomic burden [5].

With regard to the Italian setting, a study conducted in the Veneto Region, using clinical fundus examination, confirmed literature data with a prevalence of DR of 26.6% [6]. A significant worldwide increase in the number of persons affected by diabetes has been estimated for the next 10 years, reaching approximately 380 million by 2025, mostly in developing countries. In Europe, compared with 2007, there is expected to be an increase of 10 million persons with a prevalence of diabetes below 10% [7].

As the prevalence of diabetes is expected to rise in the future, an associated increase in DR cases should also be expected. However, recently, data suggest that the prevalence of DR may decrease, due to the intensification of the screening programmes and better control of risk-factors, thus underlying their effectiveness and the significance [8]. In this view, scholars and practitioners widely agreed that DR, at any stage of progression, requires specific management: from the screening programmes (useful for an early diagnosis) to the definition of pharmacological treatments needed. In recent years, the strategies for DR prevention have moved from the traditional ophthalmological examination to a faster digital retinal imaging acquisition and grading of DR. The application of these new screening programmes, followed by prompt diagnosis and a better timely management, is well known to prevent significantly the risk of diabetic blindness [9, 10], as well as to decrease all the costs related to the investigated pathology. In fact, from an economic point of view, the annual cost per patient affected by DR is approximately twice as high as those patients with diabetes only [11]: thus, the implementation of effective and recognized screening programmes could represent cost-effective strategies [12, 13], one useful to narrow both the economic and the social burden of DR.

Despite the implementation of successful national screening programmes worldwide, the lack of diffuse screening activities for DR in Italy is delaying the diagnosis and prompt treatment of DR.

Public screening programmes and prompt treatments have been shown to reduce lifetime costs related to visual disability [14, 15]. For these reasons, vision impairments as a result of DR should be considered easy to prevent, and the development of systematic programmes of screening should represent an urgent and primary healthcare need.

Moving on from these premises, since DR should be one of the major priorities for the healthcare services, the present study aimed at investigating the feasibility related to the introduction of a specific and accurate screening programme in the catchment area of Treviso (defined as ULSS 9), in comparison with the “no prevention” strategy, including also the costs averted to blindness, in terms of the validity of the intervention and the direct costs absorbed (efficiency) by the regional healthcare service. The analysis was designed assuming the regional healthcare service (Veneto Region) point of view.

## 2. Materials and Methods

### 2.1. Study Design

In order to achieve the previously mentioned objective, a perspective study for the implementation of a screening programme devoted to DR was planned to be performed between September and December 2012, after receiving the approval from Ethic Committee of the Local Health Authority. As DR is a multiprofessional and a multidimensional eye disease, a multidisciplinary group was involved in the project, including general practitioners, diabetes experts, administrative staff, nurses, epidemiologists, and ophthalmologists. The involvement of different healthcare professionals in the screening programme could influence the resources absorption and the optimisation of the screening pathway [12, 13, 16–18].

The screening involved all types (type 1 and type 2) of diabetic patients living in the area of Ponzano (Treviso) that has 5.000 inhabitants and is part of one of the Local Health Authorities of the Veneto Region: the ULSS 9 of Treviso. Diabetic subjects were accurately identified by crossing different databases: primary and secondary diagnosis for hospitalisation, drugs and delivery systems, prescriptions, health care procedures covered by the diabetic code, and the diabetic code assigned to patients. In particular, the following patients were invited to attend the screening programme: (i) outpatients going to a hospital specialist visit to the diabetologists, or first visit to ophthalmologist; (ii) patients going to the territorial pharmacist, in order to retrieve the specific diabetes drugs; and (iii) patients referring to the general practitioners, with a diagnosis of diabetes. Individuals who were already diagnosed with DR were not included in the study.

Initially, a personal letter of invitation was sent to all the diabetic target population (*N* = 498) to be enrolled in the screening programme. All the patients attending the screening procedure have signed a specific informed consent form, consistent with ethical aspects. Screening for diabetic retinopathy was made using a nonmydriatic fundus camera. A semiobscured visiting room was used to optimise physiological mydriasis before each exam. Three nonmydriatic, 45° field, digital retinal images were captured, in accordance with a previously validated technique. The three fundus images encompassed the following retinal fields: field 1 centred on the macula, field 2 centred on optic disc, and field 3 midperipheral superior-temporal field [19]. The images were obtained by trained paramedical staff (in particular, nurses). All images were electronically transmitted to the reading centre and stored in an online secured database called “Eye Knowledge Network” for the second step, online examination. Retinal images were graded for DR at the Ca' Foncello Treviso Hospital, where the Ophthalmology Unit is located, by two experts and certified readers who were members of the Reading Centre staff of the University of Padova. DR and diabetic macular edema (DME) were graded in accordance with the International Classification proposed by the American Academy of Ophthalmologists [20]. When the quality of the images was “inadequate” for the clinical evaluation and when fundus images were graded as “positive,” the patients were referred for further ophthalmologic examination. “Positive” findings included retinal changes that required specialist management: moderate and severe nonproliferative DR, proliferative DR, DME, and/or any other retinal abnormality. Further ocular and diagnostic examinations or treatments, if necessary, were then planned. Fundus images were graded as “negative” if no DR or nonsight threatening DR was detected. A report, with the results of the screening and the correct follow-up timetable for the “negative” screened population, was sent to the patient's general practitioner within 1 month after the screening.

### 2.2. Economic Evaluation

From a methodological point of view, in order to quantify the impact of the introduction of screening programme in the clinical practice, both an activity based costing (ABC) analysis and a budget impact analysis (BIA) were conducted.

The ABC [21, 22] is useful for the enhancement of the average costs related to each phase of the screening pathway. In particular, the main objective of the ABC is the measurement of the costs and the performance activities, taking into account also the related human and materials resources for the proper development of the procedure. In this view, the economic impact of each patient was determined utilising the following components: (i) human resources (i.e., individuals involved in the different phases of the screening programme, such as administrative staff, nurses, and ophthalmologists); (ii) materials and equipment; (iii) pharmacological and/or laser treatment. The ABC did not take into consideration the costs related to the delivery of the invitation letter: different technologies could be implemented in order to carry out this task (telephonic invitation, e-mail contact, personal contact, or letter), differing for the related economic value.

After the implementation of the ABC, the BIA was applied. A BIA allows the prediction of the potential financial impact of a new technology adoption, into a healthcare system [23]; influencing, in a positive or in a negative manner, the healthcare expenditure and considering both a specific point of view and a determined time horizon. In this view, two scenarios were simulated, thus comparing the so-called “do nothing strategy” with the implementation of a proper screening programme. In particular, in both scenarios, the occurrence and the related cost of blindness were taken into consideration as direct healthcare costs. Since the analysis assumed the Healthcare Regional Services point of view, the intangible and indirect items of expenditure were excluded from the study.

The economic analysis used the 2015 Italian Outpatients and Hospital Admissions Reimbursement Tariffs. Drug costs were derived from the officially published NHS price list. If necessary, economic values were reported in “euros,” considering the 2015 inflation rate, using the Consumer Price Index for healthcare expenditure, thus making economic measures comparable, and being based on the same year of reference.

In order to ensure the robustness of the result, a sensitivity analysis was carried out. In particular, both the attendance rate to the screening programme and the percentage of patients undergoing the complete eye examination were modified, thus understanding if significant changes in the feasibility of the programme occur.

Literature reported that patients' compliance with DR screening is not optimal worldwide, hence reporting attendance rates ranging from a minimum of 32% and a maximum of 92% [24–27]. Further analysis reported a variation in the eye examination rate. The Local Health Authority involved declared that it could reach about the 80%–85% if collaboration with community-based organisations, as well as greater information activities of DR risks among citizens, is implemented.

Moving on from these premises, 4 different analyses were performed.

## 3. Results

### 3.1. The Sample under Assessment

498 individuals were identified as being diabetic patients within the area of Ponzano (that has 5,000 inhabitants); thus, it emerged that the prevalence of the diabetes in the investigated town was around 10%.

Out of the 498 diabetic patients originally invited to enroll in the screening programme, 340 accepted to be evaluated (68%), although it was possible to confirm a response rate equal to 80% since 57 patients did not attend the screening programme because they had already undergone a complete eye examination that showed no signs of DR development. No patients with a previous DR diagnosis were enrolled in the present study.

The study population was composed predominantly of males (55%); the average age of the sample was 68 years (range: 26–93). On average, patients have been suffering from diabetes for 20 years (range: 2–41). The most common comorbidities developed by patients were hypertension (40%) and dyslipidaemia (38%). 324 patients (95%) successfully completed the procedure, though in 16 cases (5%) images were noncaptured due to either systemic conditions of the patients, insufficient mydriasis, or other technical reasons related to the fundus camera. The quality of digital images was adequate for the interpretation in 260 patients (80%), although it was ungradable in 64 cases (20%) due to insufficient dilation, media opacity, poor fixation, or the absence of one of the captured fields.

As a result, taking into account the gradable 260 patients, 225 were classified as “negative” (87%) and 35 as “positive” (13%). Based on the entire screened population, 115 patients (34%) were referred to an ophthalmologist. Of these, 16 (14%) were patients from whom it was impossible to obtain images, 64 (56%) had no evaluable images, and 35 (30%) were “positive” cases.

Of the previously mentioned 115 patients, 92 (91%) underwent a full ophthalmological examination, giving the following results: in 67 cases (73%), DR was not detected, though 25 patients (27%) presented signs of DR. In particular, 24% (*N* = 6) had mild nonproliferative DR, 52% (*N* = 13) had moderate nonproliferative DR, 12% (*N* = 3) had preproliferative DR, and 12% (*N* = 3) had proliferative DR. Concomitant DME was present in 36% (*N* = 9) of these patients.

In 9 cases (36%), prompt treatment with intravitreal injections and/or laser photo-coagulation was required for proliferative and severe preproliferative retinopathy or macular edema.

### 3.2. Results from the Study Conducted in Ponzano

After these general remarks, regarding the screened population, an economic evaluation was required in order to investigate the amount of resources dedicated for this specific innovative programme.

It should be noted that the Veneto Region 2015 tariffs were taken into account in order to investigate the value of the complete screening programme, thus also including the entire cycle of intravitreal injections.

With reference to the abovementioned distribution of the patients, the economic resources absorption, with regard to the whole population screened pathway, was divided into four distinct and logical phases:

- 340 patients attended the screening programme, involving 1 healthcare professional (nurse) who spent 12 minutes per patient and considering the equipment amortization.
- 324 patients completed the procedure, after which 1 ophthalmologist interpreted their digital images, spending about 5 minutes per patient, considering also the workstation and the administrative staff costs.
- out of the 115 patients referred for a complete examination, only 92 underwent an ophthalmology examination, in order to obtain an in-depth analysis of the disease (in the third phase, it is important to note that the costs of all the materials and the drugs utilised by the clinicians for the complete eye examination are included in the “first visit” reimbursement tariff).
- Nine patients, who were suffering from a severe stage of pathology, received pharmacological and/or laser treatment, thus considering that both the human resources and the materials/equipment costs are included in the procedure costs, as detailed in Table 2.

It was first necessary to evaluate the costs related to the human resources involved in the screening programme. Thus, the gross monthly salary, related to each specific professional title, was taken into consideration. In particular, its time value per minute was multiplied by the time dedicated to each procedure.

Table 1 shows the categories of cost impacting on each phase and the total cost per phase.

Table 1 shows that the fourth phase absorbed the most part of the economic resources, presenting the economic evaluation of the treatment for 9 patients. In particular, three groups of patients suffering from RD or DME were considered: (i) 4 patients (45.5%) received a therapeutic cycle of intravitreal injections with Ranibizumab alone; (ii) 3 individuals (30%) received laser treatment as a support of Ranibizumab injection; and (iii) 24.5% (2 patients) received Dexamethasone, thus being consistent with literature and real-life data [28]. Treatment costs were related to a period of 12 months, in which clinicians administered to the patients a cycle composed of, on average, 3.61 (Ranibizumab) or 1.3 (Dexamethasone) intravitreal injections, based on the observation of the clinical pathway of the nine patients under investigation in the health authority of reference and consistent with other national and international literature evidence [28, 29]. No surgical interventions were performed in the observed population.

Costs for an intravitreal injection were distinguished as follows: (a) the cost of the specific drug (€644.73 for Ranibizumab and €951.75 for Dexamethasone) and (b) the cost of the procedure carried out by the health authority of reference (€290.00, independently of the administered drug). Patients performing laser therapy absorbed an additional cost equal to €81.28 for every single procedure.

The costs related to these two treatment phases are detailed in Table 2.

With reference to Tables 1 and 2, the amount of all the screening costs for patients within the area of Ponzano was equal to €35,899.81, treating the 340 patients who attended the programme.

### 3.3. Results from the Economic Evaluation Testing the Feasibility of the Screening Programme in the Grater Treviso Catchment Area

After the costs of the screening programme phases had been calculated, the prevention activity was then extended to the Treviso catchment area (considering the whole ULSS 9), in order to estimate the feasibility of the screening procedure in a larger population.

This area presents 22,000 estimated diabetes cases. If the response rate was the same as that in the pilot study (80%), 17.600 patients would be expected to attend the screening programme within the first year. Considering an average work shift of 7 hours per day, a single nurse, completely devoted to the screening activities, could be able to perform about 7,000 procedures per year. With regard to the ophthalmologist and the administrative staff, assuming an average work shift of 8 hours, it emerges that the clinician could produce on average 9,600 medical reports per year, and the administrative staff could generate a maximum amount of 19,200 documents per year. As a result, 3 members of the paramedical staff (nurses), 2 ophthalmologists, and 1 administrative would also be required to treat the abovementioned attending patients.

Investment in the required equipment would be made to cover the need of the expected diabetic citizens; in particular, it should be allocated to 5 pieces of equipment, thus requiring an overall investment of €72,000.

Considering the incidence of blindness as a result of DR (0.002% per year, taking into account the whole ULSS 9 that has 419,728 inhabitants), 8 new cases every year would be expected if the screening programme was not applied. The screening programme conducted reported an effectiveness equal to 74.59%, calculated as the difference between individuals attending the screening programme and individuals who did not participate in the prevention activities, or patients who denied to go to the ophthalmologist for the complete eye examination. Considering the Treviso catchment area, 4,400 individuals did not attend the screening programme whereas 1,190 did not perform the in-depth visit, thus reaching a total amount of 5,590 individuals (25.41% of the overall invited population). In this view, in the innovative scenario, only 2 patients would develop blindness.

Literature evidence [30] shows that the cost of blindness is approximately $18,670 (i.e., €16,803), including only medical and direct costs.

In addition, therapeutic treatment should be administered to 8% of DR patients, considering both the screening pathway and the “no prevention” strategy. In particular, 381 patients referring to the screening pathway scenario and 609 patients (thus considering a DR occurrence rate equal to 34.60% [31] within the diabetic population) for whom “no prevention” strategy was implemented received drugs therapy. The distribution of the treated population, considering the administered drug (74.5% for Ranibizumab alone or with the support of laser therapy and 25.5% for Dexamethasone [28, 29]), as well as the treatment frequency within a 12-month time horizon, was assumed to be the same as the conducted study previously described.

With reference to these data, the total costs of the two different pathways are presented in Table 3. In this view, it emerged that in both scenarios the more significant economic resources absorption was related to the treatment phase: at 12-month time horizon, the administration of Ranibizumab with the support of laser therapy, Ranibizumab alone, or Dexamethasone required, on average, €3,667.80, €3,374.38, and €1,614.28, respectively, per patient.

Table 4 reports the results of the sensitivity analyses, demonstrating an overall economic advantage in all the cases in which the prevention activity is implemented, thus ensuring the robustness of the BIA result. In particular, it emerged that the economic benefits of the screening programme implementation are more sensitive to the higher number of screened individuals, in comparison with the decrease of the blindness events that occurred.

More investments are required from the healthcare service, if the screening programme would cover a larger number of the target population: in particular, achieving a maximum attendance rate equal to 92%, the healthcare service would equally benefit of an economic saving of −2.38%, always resulting in the preferable solution.

## 4. Discussion

Screening for diabetic retinopathy is important because the majority of patients who develop DR show no symptoms until diabetic macular edema and/or proliferative diabetic retinopathy are present, thus confirming that, in the early stages of the investigated disease, any notable symptoms affect the patients.

Although the beginning of the DR has an asymptomatic nature, the attendance rate reported in the proposed study was equal to 80%, thus demonstrating the effectiveness and the validity of the screening programme. An increase in the attendance rate could be achieved with an improvement and a diversification of the communication tools to inform patients concerning the importance of the eyes examinations and controls, thus enhancing their awareness on this field. In particular, literature [32] reported that telehealth or telemedicine programmes may facilitate early DR diagnosis and timely treatment, preserving vision.

The results of the study show the importance of a screening programme, from an economic point of view, one leading to a substantial saving of €271,543.32 (−13.71%) in comparison with the “no prevention” strategy. In addition, three-field colour, 45-degree, nonmydriatic images have demonstrated a sensitivity and specificity of 82% and 92%, respectively, in the diagnosis of DR, representing an effective tool in a screening setting [19].

Traditionally, ophthalmologists evaluate patients for DR by mydriatic indirect ophthalmoscopy. The references for the correct follow-up and management are the AAO and national guidelines for DR [33, 34]. However, despite the relevance of implementing a screening programme, in the real world, these recommendations are seldom adhered to. The increasing number of diabetic patients, in particular due to the population ageing, delays access to the next ophthalmologic examination. It has been estimated that only 50% of the known diabetic patients receive the recommended regular eye examinations [35], something that may be considered a real concern. Eye screening offers the possibility of identifying the early signs of DR, thus preventing visual loss due to DME. As previously described, new technological screening procedures based on digital mydriatic and nonmydriatic fundus images present multiple advantages. Images can be taken by trained nonspecialist operators and can be viewed “online” by specifically trained ophthalmologists in a deferred time, thus sparing and optimising resources. This could lead to a significant benefit with the decreasing of waiting lists, a phenomenon perceived by citizens as a serious problem of modern healthcare systems that compromises the coverage of their health needs. In addition, because of their easy and safe use, without the administration of drugs, nonmydriatic cameras can be placed in primary care settings in order to improve the access to care.

A large-scale application of this screening strategy could spare unnecessary examinations for “negative” patients, thus preventing irreversible loss of visual acuity for persons affected by retinopathy, due to long waiting lists for eye examination.

In particular, it emerged that the screening programme presented in the study has the potential to reduce the prevalence of blindness due to DR in the Veneto Region.

With reference to this, the nonmydriatic fundus camera is not only effective but also cost-effective in the investigated greater area and leads to significant benefits for both the regional healthcare service and for the patients. Vision loss is associated not only with a large increase of costs due to the management of this condition but also with the compromise of a patient's quality of life, thus representing a significant social burden and requiring a future in-depth analysis. In fact, with the rapid ageing of the population, and considering that older adults could stay active and also productive in this specific part of their lives, blindness should be a public healthcare priority.

Healthcare regulators and policy makers will benefit from the implementation of adequate screening programmes, optimising effectiveness and resources allocation within this specific target population. In this view, the present study would represent the first attempt to extent the current theories and models into the practical context of the healthcare sector, extending the results of the study, from the Italian setting to other European and international contexts. The results would contribute in the advancement and establishment of organisational and management models to be applied in the prevention sector, freeing up hospital resources for more severe cases of patients and reducing the economic and social burden of waiting lists for ophthalmic procedures. The early diagnosis and the process of taking charge of the patients could be considered an effective way to offer a better healthcare delivery to the patients, a clinical pathways optimisation but, in particular, a possible economic saving for the healthcare services.

A future interesting contribution for policy makers could be also the definition of the best screening programme organisational setting, maximizing both effectiveness and efficiency, involving different healthcare professionals, such as general practitioners and pharmacists. New approaches to the screening development could reduce healthcare expenditure and increase the attendance rate for patients.

In this view, further researches could be addressed to the proposal of a reimbursement tariff for the diabetic screening activities, with reference to different clinical setting in which the procedure could be performed, as well as the implementation of a multidimensional assessment (through the health technology assessment tool) in order to understand all the possible implications (organisational, social, and equity aspects) of the adoption of screening programmes, measured into the clinical practice, generating significant advancement to these findings and their robustness.

## Figures and Tables

**Table 1:** Cost of the screening programme, distinguished by phases.

Phases	Human resources	Materials and equipment	Drugs	Total
Phase I	€1,056.23	€2,112.46	—	€3,168.69
Phase II	€1,170.98	€1,170.39	—	€2,341.37
Phase III	€3,109.10	—	—	€3,109.10
Phase IV	—	—	€27,280.65	€27,280.65
				*Total *€*35,899.81*

**Table 2:** Details of the treatment's costs.

Treatment option	Cost of the drug administered	Procedure	Total cost for a single injection	Total cost for a therapeutic cycle	Total cost for the treated population (9 patients)
Ranibizumab + laser therapy	€644.73	€371.28	€1,016.01	€3,667.80	€9,903.07
Ranibizumab	€644.73	€290.00	€934.73	€3,374.38	€22,928.93
Dexamethasone	€951.75	€290.00	€1,241.75	€1,614.28	€3,559.48

**Table 3:** Economic resources related to the investigated procedures.

Screening programme pathway	
Phase I	€164,026.11
Phase II	€121,230.90
Phase III	€160,924.94
Phase IV (treatment)	€1,154,724.70
Investment in equipment	€72,000.00
Blindness	€35,843.46
*Total*	€*1,708,750,11*

“Do nothing” strategy	

Blindness	€134,424.00
Treatment	€1,845,869.43
*Total*	€*1,980,293.43*

*Economic savings*	*−*€*271,543.32*
	*−13.71%*

**Table 4:** Sensitivity analyses.

Sensitivity analysis	Screening attendance rate	Eye examination rate	“To do nothing” strategy	Screening programme pathway	Difference (€)	Difference (%)
Sensitivity analysis I	32%	75%	€1,980,293.43	€811,332.32	−€1,168,961.11	−59.03%
Sensitivity analysis II	92%	75%	€1,980,293.43	€1,933,104.55	−€47,188.87	−2.38%
Sensitivity analysis III	80%	85%	€1,980,293.43	€1,789,070.03	−€191,223.39	−9.66%
Sensitivity analysis IV	80%	90%	€1,980,293.43	€1,869,389.96	−€110,903.47	−5.60%
